# Anti-*Onchocerca* and Anti-*Caenorhabditis* Activity of a Hydro-Alcoholic Extract from the Fruits of *Acacia nilotica* and Some Proanthocyanidin Derivatives

**DOI:** 10.3390/molecules22050748

**Published:** 2017-05-06

**Authors:** Jacqueline Dikti Vildina, Justin Kalmobe, Boursou Djafsia, Thomas J. Schmidt, Eva Liebau, Dieudonne Ndjonka

**Affiliations:** 1 Department of Biological Sciences, Faculty of Science, University of Ngaoundere, P.O. Box 454, Ngaoundere, Cameroon; jdikti@yahoo.fr (J.D.V.); jkalmobe@yahoo.com (J.K.); vboursou1@googlemail.com (D.B.); 2 Institute of Pharmaceutical Biology and Phytochemistry (IPBP), University of Münster, PharmaCampus Correnstrasse 48, D-48149 Münster, Germany; thomschm@uni-muenster.de; 3 Institute for Zoophysiology, University of Münster, Schlossplatz 8, D-48143 Münster, Germany; liebaue@uni-muenster.de

**Keywords:** onchocerciasis, *Acacia nilotica*, *Caenorhabditis elegans*, *Onchocerca ochengi*, proanthocyanidins

## Abstract

*Acacia nilotica* fruits with high tannin content are used in the northern parts of Cameroon as anti-filarial remedies by traditional healers. In this study, the hydro-alcoholic fruit extract (crude extract (CE)) and, one of the main constituents in its most active fractions, (+)-catechin-3-*O*-gallate (CG), as well as four related proanthocyanidins, (−)-epicatechin-3-*O*-gallate (ECG), (+)-gallocatechin (GC), (−)-epigallocatechin (EGC) and (−)-epigallocatechin-3-*O*-gallate (EGCG), were assessed for their potential in vitro anthelmintic properties against the free-living model organism *Caenorhabditis elegans* and against the cattle filarial parasite *Onchocerca ochengi*. Worms were incubated in the presence of different concentrations of fruit extract, fractions and pure compounds. The effects on mortality were monitored after 48 h. The plant extract and all of the pure tested compounds were active against *O. ochengi* (LC_50_ ranging from 1.2 to 11.5 µg/mL on males) and *C. elegans* (LC_50_ ranging from 33.8 to 350 µg/mL on wild type). While high LC_50_ were required for the effects of the compounds on *C. elegans*, very low LC_50_ were required against *O. ochengi*. Importantly, tests for acute oral toxicity (lowest dose: 10 mg/kg) in Wistar rats demonstrated that crude extract and pure compounds were non-toxic and safe to use. Additionally, the results of cytotoxicity tests with the Caco-2 cell line (CC_50_ ranging from 47.1 to 93.2 µg/mL) confirmed the absence of significant toxicity of the crude extract and pure compounds. These results are in good accordance with the use of *A. nilotica* against nematode infections by traditional healers, herdsmen and pastoralists in Cameroon.

## 1. Introduction

Onchocerciasis (river blindness) is a tropical insect-borne disease caused by the nematode *Onchocerca volvulus* and transmitted through the bite of infected black flies (*Simulium* spp.). It is classified by the World Health Organization (WHO) as one of the tropical neglected diseases (NTDs). It is widespread in Sub-Saharan Africa and endemic in 19 African countries with some foci in Yemen and parts of Latin America. The disease is mainly characterized by skin irritations and eye lesions and can ultimately lead to blindness [1]. It has been estimated that 36 million people are infected [2], and 86 million people live in high risk areas of the African Programme for Onchocerciasis Control (APOC) countries. Onchocerciasis is responsible for about 270,000 cases of blindness and 500,000 cases of visual impairment [1]. Furthermore, people with this disease lose their productivity, thereby affecting the economy of the endemic countries.

Control strategies currently in use rely on mass administration of ivermectin (Mectizan). While this drug has marked activity against microfilariae, it is much less effective against adult worms. In Cameroon, ivermectin was reported to induce some severe inflammatory reactions in people co-infected with loasis [3]. Furthermore, the development of resistance to ivermectin was observed in some communities in Ghana and Sudan [4,5,6]. Treatment with antibiotics, such as tetracycline or doxycycline, targeting the endobacteria *Wolbachia,* has also been reported [7,8]. However, its use is restricted to only some nematodes, since others such as *Loa loa* do not have *Wolbachia* [9]. Furthermore, continuous use of antibiotics is known to lead to the emergence of resistance [9]. Since vaccines and safe macrofilaricidal treatment against *Onchocerca volvulus* are still lacking, there is an urgent need to discover novel drugs. Natural sources, such as plants, represent a major opportunity to discover new lead molecules.

In our study, we investigated the bovine parasite *Onchocerca ochengi* that is also transmitted by *Simulium damnosum* and considered to be the closest relative of *O. volvulus.* It is currently the best model available for performing research in chemotherapy and immunology of onchocerciasis, and it is demonstrated that drugs used against the bovine parasite also affect the human parasite [10].

Research over many years has demonstrated the medicinal usefulness of many plants to combat parasitic nematodes. An online search on medicinal plants used against onchocerciasis found nine publications since 2002, where a total of 17 plant species, belonging to 10 different families, have been studied [11]. In these studies, only two pure compounds were isolated and tested on *Onchocerca gutturosa* [12]. Commercially available pure compounds, such as gallic acid, gentisic acid and ellagic acid, have been tested on *O. ochengi* and against the non-pathogenic nematode model organism *Caenorhabditis elegans* [13]. The majority of studies have shown in vitro nematocidal activities of plant extracts (*Homalium africana*, *Margaritaria discoidea*, *Anogeissus leiocarpus*, *Khaya senegalensis*, *Euphorbia hirta*, *Annona senegalensis*, *Hagenia abyssinica*, *Acer rubrum, Rosa multiflora*, *Quercus alba*) on *C. elegans* and *O. ochengi* [14,15,16,17,18,19]. Fractions from *Craterispermum laurinum* and *Morinda lucida* have shown activities with LC_50_ concentrations ranging from 7.8 µg/mL to 46.8 µg/mL on *O. ochengi*. The lowest LC_50_ values of secondary metabolites (AMJI and linoleic acid) from *Cyperus articulates* recorded were 15.7 µg/mL and 55.7 µM on the same worm [20,21,22]. Up to present, no pure compound has shown activity with an LC_50_ lower than that of ivermectin (1.7 µM) on *O. ochengi*.

*Acacia nilotica* is claimed by local traditional healers in Cameroon to be an effective anthelmintic [23]. *Acacia nilotica* (Linn) Willd ex Delile, also known under the synonyms *Acacia arabica* (Lam) var. *nilotica* (L.) Benth., *Acacia scorpioides* (L.) var. *nilotica* (L.) A. Chev., *Mimosa nilotica* Linn., is widely distributed in different regions of the world (Australia, Asia, Africa and America) [23,24]. *A. nilotica* is found in the northern part of Cameroon, and it is known in Cameroonian folk medicine by the common names of “Galbiki” or “Gavdi” in Foulfoudé and “Acacia du Nil” in French [23]. Different parts of the tree (gum, leaves, root, fruits, seeds, stem bark, branches and wood) are traditionally used in Africa for treating colds, bronchitis, pneumonia, ophthalmia, diarrhea and hemorrhage. Furthermore, *A. nilotica* was shown to have anti-bacterial, anthelmintic, antiplasmodial, anti-mutagenic, antipyretic, antioxidant and anti-hypertensive activities [25].

*Acacia nilotica* contains a variety of bioactive components, such as gallic acid, ellagic acid, isoquercetin, leucocyanidin, kaempferol-7-diglucoside, naringenin-7-*O*-β-d-(6′-*O*-galloyl) glucopyranoside, rutin, apigenin-6,8-bis-*C*-glucopyranoside, m-catechol and their derivatives, as well as galloylated derivatives of (+)-catechin and (+)-gallocatechin [26,27,28]. A literature survey on *A. nilotica* revealed anthelmintic effects against the intestinal helminth *Haemonchus contortus* [29,30].

This paper reports, for the first time, the activity of the hydro-alcoholic crude extract of *Acacia nilotica*, some of its polyphenol-rich fractions and one of its major constituents, catechin-3-*O*-gallate, against the cattle parasite *Onchocerca ochengi* and mutant anthelmintic-resistant strains of *Caenorhabditis elegans*. Furthermore, some structurally-related proanthocyanidins, namely (+)-gallocatechin, (−)-epicatechin-3-*O*-gallate, (−)-epigallocatechin and (−)-epigallocatechin-3-*O*-gallate, were also included in the tests and found active with LC_50_ concentrations in the low micromolar range. The tested compounds also demonstrated nematocidal activity against *Caenorhabditis elegans* wild type and various drug-resistant strains, but only at higher concentrations. Their cytotoxicity, as well as acute oral toxicity were also assessed.

## 2. Results

### 2.1. Dereplication of the Active Fractions and Isolation of Catechin-3-O-Gallate as the Major Constituent

**Figure 1:**
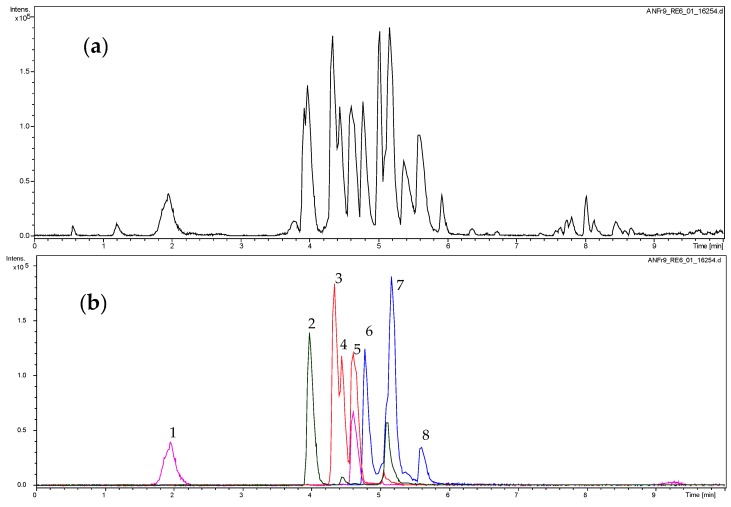
UHPLC/+ESI QqTOF MS (Ultra-high performance liquid chromatography positive electrospray ionization quadrupole/time-of-flight mass spectrometer) analysis of Fraction 9. (**a**): base peak chromatogram (*m*/*z* 100 to 1000); (**b**): extracted ion chromatograms for *m*/*z* 443 (blue), 291 (green), 458 (red), 307 (magenta). For peak assignment, see Figure 3.

**Figure 2:**
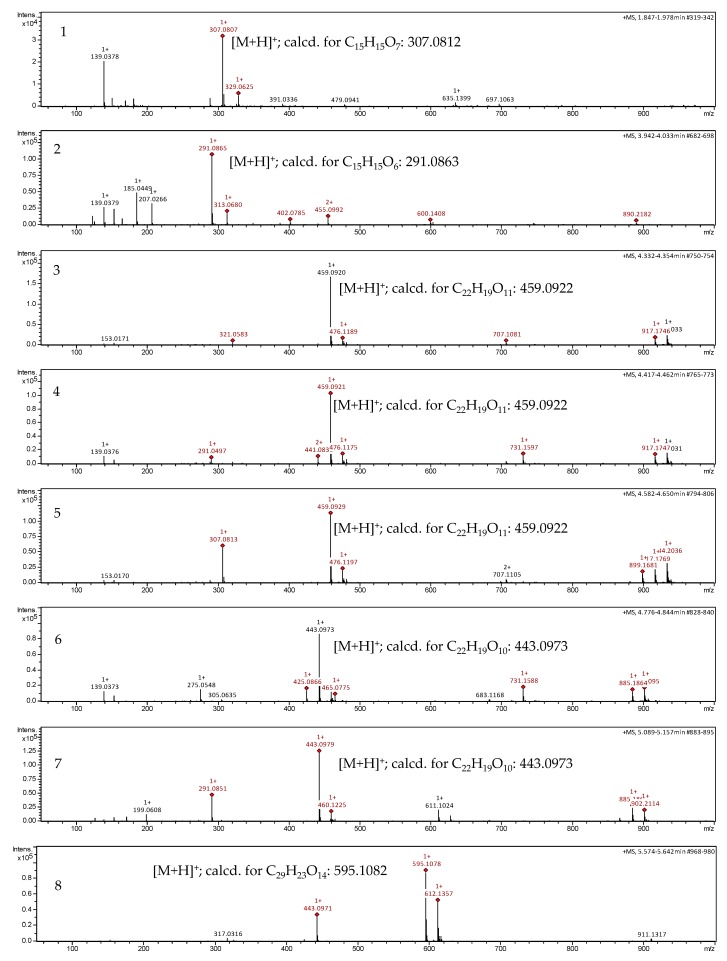
+ESI QqTOF MS spectra of Peaks 1 to 8 (see Figure 2). Assignment of spectra: (**1**) gallocatechin (GC); (**2**) catechin; (**3**) gallocatechin-3′- or 4′-*O*-gallate; (**4**) gallocatechin-7- or 5-*O*-gallate; (**5**) gallocatechin-3-*O*-gallate; (**6**) catechin-*O*-gallate (or gallocatechin-protocatechuate); (**7**) catechin-3-*O*-gallate (CG); (**8**) catechin-di-*O*-gallate. Peak 7 represents the isolated catechin-3-*O*-gallate.

**Figure 3:**
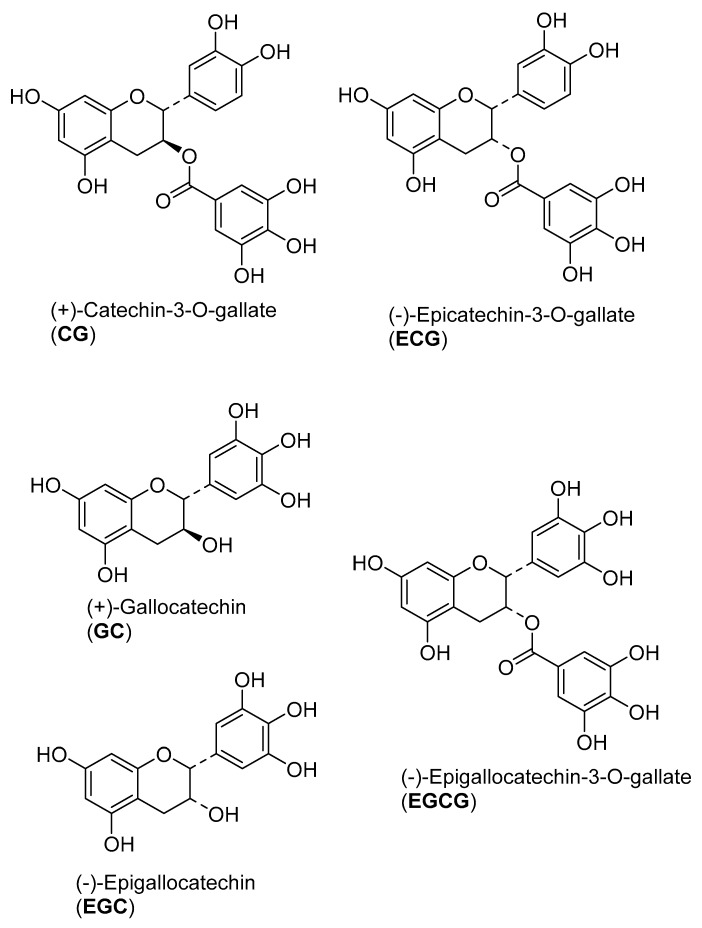
Chemical structures of the proanthocyanidins tested for anthelminthic activity.

The active fractions were analyzed by UHPLC/+ESI MSMS in order to detect and characterize their main constituents (see Figure 1 and Figure 2). They were found to contain the proanthocyanidins gallocatechin and catechin along with galloylated derivatives as could be deduced from the characteristic molecular ions and fragment mass signals obtained at high resolution that allowed unambiguous determination of the elemental formulas (see Figure 2). Besides the peaks of gallocatechin (1) and catechin (2), the fractions contained various galloyl esters of these proanthocyanidins, namely with galloylation in the A-, B and C-ring as previously reported from this plant [26], which could be distinguished by careful inspection of their mass spectral fragmentation. Thus, Peaks 3 to 5 all represent isomers of elemental formula C_22_H_18_O_11_ corresponding to mono-galloylated gallocatechin. Peaks 6 and 7 corresponded to monogalloylated catechin derivatives (elemental formula C_22_H_18_O_10_), whereas Peak 8 could be assigned a catechin digallate (C_29_H_22_O_14_). As a major component, catechin-3-*O*-gallate (CG; Peak 7 in Figure 1 and Figure 2) was isolated by preparative. HPLC (retention time = 20 min; UV: λ_max_ = 202 nm) from Fraction 8 and identified by NMR spectroscopy. Its data were identical with those published on this compound isolated from *Camellia sinensis* and *Piptadenia pervillei* [31,32]. CG was subsequently investigated for anti-nematode activity as the pure compound together with its stereoisomer epicatechin-3-*O*-gallate (ECG) and three further structurally-related compounds (gallocatechin (GC); epigallocatechin (EGC) and epigallocatechin-3-*O*-gallate (EGCG). The structures of the tested compounds are shown in Figure 3.

### 2.2. Nematocidal Activity against C. elegans

During the screening of plant extract for anthelminthic activity, the crude hydro-alcoholic extract of *Acacia nilotica* fruits (crude extract (CE)) showed activity against the free-living nematode *C. elegans* and the cattle parasite *O. ochengi* (see Table 1). The extract was fractionated by column chromatography on silica, and the 16 fractions obtained were tested against *C. elegans*. Of the 16 fractions, the first six display required higher concentrations than 1000 µg/mL; the most active were Fractions 8 to 10 with LC_50_ of 73.8 ± 0.2; 70.1 ± 0.3; 51.6 ± 0.9 µg/mL, respectively (see Supplementary Materials Table S1). These fractions were therefore selected for analysis of their constituents of which the most abundant was catechin-3-*O*-gallate.

The pure catechin-3-*O*-gallate, as well as the other related proanthocyanidins were tested for nematocidal activity using young adults of *C. elegans*. Levamisole, ivermectin and albendazole were used as the positive reference. No death was observed during the experimental period of 48 h in the negative controls. Table 1 shows the LC_50_ results of the tested compounds. Compared to levamisole (LC_50_ values of 7.2 µM), the crude extract exhibited a higher LC_50_ of 350 μg/mL after 48 h on *C. elegans* N2 Bristol WT. The crude extract therefore causes a much lower mortality than the positive control. Table 1 shows that the five proanthocyanidins tested as pure compounds exhibit nematocidal activity at LC_50_ below that of the crude extract at the same exposure period (48 h).

Table 1 shows that the crude extract is most potent on the WT strain compared to the levamisole-, ivermectin- and albendazole-resistant strains. All tested drug exhibited the mortality effect on all strains of *C. elegans* used at different LC_50_ values (Table 1).

Among the tested samples, WT is moderately sensitive to ECG (LC_50_ 2.4 µM) and EGCG (LC_50_ 108.6 µM) compared to EGC, GC and CG (LC_50_ 204.7; 242.4; 204.8 µM, respectively) (see Figure 3 for the structures of the compounds). Additionally, the ivermectin-resistant strains VC722 (LC_50_ 61.4 and 95.4 µM respectively) and DA1316 (LC_50_ 61.9 and 89.1 µM respectively) are highly sensitive to ECG and moderately sensitive to EGCG. A similar effect is observed with the albendazole-resistant strain CB3474 (LC_50_ 44.2 and 77.9 µM respectively) (Table 1).

The levamisole-resistant strain of *C. elegans* CB211 (LC_50_ 70.2 µM) is highly sensitive, while ZZ16 (LC_50_ 214.2 µM) is moderately sensitive to GC. The four other compounds are less sensitive on these two strains as shown in Table 1. Additionally, the levamisole-resistant strain CB211 is also highly sensitive to EGCG (LC_50_ 7.4 µM). Albendazole displays the highest LC_50_ (26.4 µM) compared to the two other reference drugs, while ivermectin exhibiting the lowest LC_50_ (1.3 µM). In general lethal concentrations (LC) are higher on *C. elegans* compared to *O. ochengi*. The WT is not highly active to the compounds. The isolated compound exhibited lethality at least three-fold compared to the CE (Crude extract).

### 2.3. Nematocidal Activity against Onchocerca ochengi

The compounds were also assessed for their toxicity to microfilariae (Mf), male and female *O. ochengi*. Levamisole, ivermectin and albendazole were used as positive references. As already mentioned above, the CE displayed a remarkable activity against all three forms of the parasite with LC_50_ values between 1.0 and 12.0 µg/mL.

Table 1 shows that EGCG (LC_50_ 2.6, 2.3 and 2.8 µM against male, female and Microfilariae) and ECG (LC_50_ 4.7, 4.7 and 2.2 µM, respectively) exhibit the highest mortality of the tested compounds (see Figure 3 for the structures of the compounds). The activity level of EGCG was in the same range as that of the positive control, levamisole (5.1, 10.2 and 5.1 µM, respectively). The other tested pure proanthocyanidins displayed a somewhat weaker, but still very significant, activity.

It is therefore clear that the proanthocyanidins present in the active fractions of *A. nilotica* are the constituents mainly responsible for the crude extract’s nematocidal activity.

### 2.4. Cytotoxicity against Caco-2 Cells

The results in terms of CC_50_ values shown in Table 2 demonstrate that the viability of Caco-2 cells is inhibited by the compounds. There is no difference between the CC_50_ values for GC and for those of ECG and CG because the value for GC is within the ± SD of ECG and within the ± SD of CG. The crude extract is less toxic than the conventional anthelmintics tested (Table 2). The order of CC_50_ toxicity is therefore CE > CG = ECG = GC > EGCG > EGC. Comparison of the selectivity indices (SI = CC_50_/LC_50_) calculated for *C. elegans* and *O. ochengi* (Table 2) again shows that *C. elegans* is much less sensitive to the tested chemicals than the mammalian control cells, whereas *O. ochengi* is significantly more sensitive. Generally, the SI of the crude extract and almost all compounds indicated good selective anthelminthic activity on parasites (*O. ochengi*). This shows that some selectivity of the compounds under study exists. Hence, it will justify their more detailed investigation against this parasite.

### 2.5. Acute Mammalian Toxicity

An acute toxicity study was performed to evaluate the safe administrable doses of *A. nilotica* (L.) fruit extract and isolated proanthocyanidins on albino Wistar rats for 14 days. In vivo studies revealed that no abnormal behavior and no mortality during the treatment and observation periods were observed in animals treated at the doses employed. The results of the acute oral toxicity tests of the five compounds CG, ECG, GC, EGC and EGCG, as well as the hydro-alcoholic crude extract of *A. nilotica* using a rat model are presented in Table S2. The low doses of 10, 100 and 1000 mg/kg of each drug used in Phase I and administered orally to a group of three rats led to no mortality. As no mortality was observed in Phase I, doses were increased to 1500, 3000 and 5000 mg/kg. Even at these doses, no mortality was recorded. Adverse reactions like increased motor activity, blinking eyes, tremors, convulsion, lacrimation, stimulation, muscle weakness, sedation, urination, salivation, lethargy, sleep, arching and rolling and coma up to a dose of 5000 mg/kg were not noticed within 14 days. Our results confirm that the doses tested were harmless for further in vivo investigations via gavage.

## 3. Discussion

Previous reports state that *A. nilotica* has anthelmintic activity against *Haemonchus contortus* and suggest the lipophilic nature of the active principle in bark and leaves [33]. In a separate study, a variety of bioactive components such as gallic acid, ellagic acid, isoquercetin, leucocyanidin and kaempferol have been isolated from *A. nilotica* [28,34]. Even though some of these phenolic compounds obtained from other sources have been tested against *O. ochengi* [13], as far as could be ascertained, this is the first report on the anthelminthic activity of CG, isolated from the fruit of *A. nilotica*, and on its structural congeners, ECG, GC, EGC, EGCG as individual compounds on this bovine parasitic nematode, *O. ochengi*, and the mutant anthelmintic-resistant *C. elegans*.

This study was undertaken to assess the anthelmintic efficacy of the crude extract of *A. nilotica*, as well as its major constituent CG against the bovine filarial nematode *O. ochengi* and the free-living nematode *C. elegans*. Some further related proanthocyanidins, ECG, GC, EGC and EGCG, were also included in the study because of their chemical similarity to the *A. nilotica* constituents. The nematodes under study have widely been used to evaluate the efficacy of several anti-filarial agents [20,21,22,35,36]. In this study, the toxicity of the extract and the pure compounds was investigated against *C. elegans* WT and five drug-resistant mutant strains (ZZ16, CB211, CB3474, VC722, DA1316). Furthermore, the activity against two different life cycle stages (macrofilariae and microfilariae) of the cattle parasite *O. ochengi* was assessed. Our results demonstrate the sensitivity of both nematodes to the pure compounds; the parasite being significantly more sensitive than the free-living nematode. Although there are many reports from Asia, Africa, America and Europe on natural plant extracts or plant-derived compounds that have anthelmintic properties, no major commercial product based on these natural compounds has been developed yet for wide use [19,37,38]. *A. nilotica* has been reported to be a rich source of tannin [26,27,28,29,30]. Condensed and hydrolysable tannins were shown to have anthelmintic potential with various degrees [13,15]. The crude extract tested in this study exhibited LC_50_ values (449 and 350 μg/mL), at 24 h and 48 h post-treatment, respectively (result at 24 h not shown), less than those reported in the literature on other plants rich in tannin, as well: *Quercus alba* (0.75 mg/mL), *Rhus typhina* (0.65 mg/mL) *Acer rubrum* (1.03 mg/mL) and *Rosa multiflora* (2.14 mg/mL) against *C. elegans* at 24 h post-treatment [15].

Our experiments showing an LC_50_ = 108.6 µM of EGCG on *C. elegans* wild type are in contrast with in vitro evidence of increased lifespan of *C. elegans*. In 2009, Zhang et al. reported that EGCG enhances the resistance of *C elegans* to environmental stress and prolongs its longevity by removing reactive oxygen species (ROS) when administered on *C. elegans* fed with *E. coli* OP50. However, in that study, the authors exposed two-day-old adult wild type to *E. coli* containing the compound on solid medium at the highest concentration of 10 µg/mL; EGCG did not extend the longevity of WT under normal culture conditions, but led to an increase in stress tolerance [39]. In contrast to these authors, we used much higher concentrations of EGCG and incubated worms without OP50 as the source of food. All five pure proanthocyanidins showed higher mortality on WT (LC_50_: ECG 2.4; EGCG 108.6; EGC 204.7; GC 242.4; CG 204.8 µM) compared to the crude extract (LC_50_ 350 μg/mL). Proanthocyanidins vary in the number of phenolic hydroxyl groups and in their stereochemistry. The difference in the efficacy of the compounds, in this study, may be due to trihydroxylated aromatic groups, i.e., the galloyl ester group and a pyrogalloyl type B-ring. Each of the five tested compounds harbored either a pyrogalloyl type B-ring or a galloyl group, but only EGCG has both groups, which might explain its high toxicity, it required higher LC_50_ values than ECG. Mukai et al. [40] also reported that EGCG toxicity surpasses mebendazole. It was also shown in the same study that the pyrogalloyl type B-ring of EGC and EGCG enhances the toxicity compared to ECG. Similarly, the galloyl group of ECG and EGCG also has great significance in causing toxicity [40]. In another study, the toxicity of gallotannins and condensed tannins towards *C. elegans* has been shown to be dependent on the degree of galloylation and polymerization, respectively [41]. The two isomers CG and ECG have different lethality on the worms. The first has the highest LC_50_ values on WT and parasite, which cannot be explained by the galloyl group. It might be that the stereochemistry of the proanthocyanidin core plays an important role.

Tannins are known to bind to free proteins in the gastrointestinal tract of host animal or glycoprotein on the cuticle of the parasite [42] and cause death. Here, we assess whether the compounds’ mode of action might be similar to those of the three classes of the most used anthelmintics: avermectins, which are glutamate-gated chloride (GluCl) potentiators, imidazothiazoles, which are nicotinic agonists, and benzimidazoles, which are ß-tubulin binding [42]. Our results revealed a varying lethality of the five resistant *C. elegans* strains to the compounds under study. CB211 and ZZ16 are knockout mutants of the genes *lev-1* and *lev-9*, respectively. The gene *lev-1* is expressed in body wall muscle and is important for normal locomotion and regulation of egg-laying [43]; the gene *lev-9* is also secreted in muscle cells and is responsible for locomotion and egg-laying. Compared to the WT (LC_50_ 74.3 μg/mL), GC displays mortality of half of the population on the levamisole resistant strains (ZZ16 and CB211) at lower LC_50_ 214.2 and 70.2 µM, respectively. Only mutant CB211 is sensitive to EGCG (LC_50_ 57.4 µM) (Table 1). This suggests that their efficacy is independent of the levamisole resistance and that the mode of action of the compounds differs from that of levamisole. Levamisole belongs to the imidazothiazoles, which are nicotinic receptor agonists [44,45]. However, mutants CB211 and ZZ16 showed a reduced sensitivity towards CG, ECG and EGC (Table 1), suggesting that the effects of these three compounds might be similar to levamisole. CB3474 is a knockout mutant of *ben-1*. This gene encodes β-tubulin that represents the binding site of albendazole, inhibiting the formation of microtubules [46,47] and resulting in the paralysis of the worms [48]. Albendazole is one of the benzimidazole carbamates [44,45]. Compared to WT, the albendazole-resistant strain (CB3474) is highly sensitive to ECG (LC_50_ 44.2 µM) and EGCG (LC_50_ 77.9 µM), suggesting that the mode of action of these compounds differs from that of albendazole. However, contrary to ECG and EGCG, the mutant CB3474 showed a reduced sensitivity towards GC, CG and EGC (Table 1), suggesting that these three compounds may act similarly to albendazole. 

Ivermectin is a drug classified amongst the macrocyclic lactones [45]. It is a GluCl receptor potentiator [49]. It specifically binds to GluCl channels and selectively paralyzes the parasite by increasing muscle and nerve chloride-ion permeability, thereby causing the death of worm [44]. VC722 is a single mutant in which the GluCl subunit *glc-2* has been knocked out. *Glc-2* represents the binding site of ivermectin in pharyngeal muscle cells [50]. DA1316 is a triple mutant in which genes *avr-14*, *avr-15* and *glc-1* are knocked out. Genes *avr-14* and *glc-1* represent the binding site of ivermectin in the nervous system, while the gene *avr-15* plays the same role as *glc-2* [50,51]. Compared to WT, the single (VC722) and triple (DA1316) ivermectin-resistant strains are highly sensitive to ECG (LC_50_ 61.4; 61.9 µM, respectively) and moderately sensitive to EGCG (LC_50_ 95.4; 89.1 µM, respectively) (Table 1). This suggests that their efficacy is independent of genes transferring resistance to the strains and may be due to the chemical structures of both molecules, which are almost identical, except for one additional OH on EGCG. Since genes conferring resistance in both mutants are in muscle cells and nervous system, high activities of EGCG and ECG observed on mutants VC722 and DA1316 could be explained by the fact that these two compounds act neither in muscle cells nor in nervous system, but in another tissue. The stereochemistry of the proanthocyanidin core appears to play an important role. Compounds of the epi-series (B-ring and Oxygen at C-3 cis-oriented), such as ECG and EGCG, appear more active than compounds with trans-orientation of these structure elements (CG and GC). Taken the high sensitivity of the ivermectin resistant strains to ECG and EGCG, their mode of action differs from that of ivermectin. Mutants VC722 and DA1316 show only a slightly reduced sensitivity towards GC, CG and EGC (Table 1). This might suggest that these three compounds do not act similarly to ivermectin. The five isolated proanthocyanidins thus appear to have a mode of action different from those of the commonly-used anthelmintic, ivermectin. However, they might act synergically with gallic and ellagic acid in the crude extract since their nematotoxicity has been shown [13]. It has long been known and demonstrated in various studies that tannins and other polyphenolic compounds are protein coagulants, which could result in a broad spectrum worm killing activity [52,53]. Iqbal et al. [54] suggested that condensed tannins may also bind to the cuticle of larvae, which is rich in glycoprotein according to Thompson and Geary [55], and cause death [54].

Results obtained after the exposure of *O. ochengi* to the five compounds reveal strong mortality. This result is in agreement with a recent study that showed that hydrolysable tannins, such as gallic acid and ellagic acid, have high levels of anthelmintic activity [13]. The present results are in agreement with these findings, since ECG and EGCG are galloylated proanthocyanidins, which contain a gallic acid moiety in their structure. Compared to the high concentrations required for the efficacy of the compounds on *C. elegans*, considerably lower concentrations are required for efficacy on *O. ochengi*. This appears to be in agreement with the statement of Geary et al. [35] on the disadvantage of using *C. elegans* as a model to estimate the intrinsic potency of anthelmintics against parasitic nematodes. The presence of galloylated proanthocyanidins in the most active fractions of *A. nilotica* extract rationalizes the use of this plant in folk medicine. The high level of activity of the tested compounds of this type against *O. ochengi*, which is in the same range as that of the positive controls, makes this class of plant phenols very promising candidates for further detailed studies.

The acute toxicity assay carried out with the drugs at a dose of 5000 mg/kg caused neither behavioral changes nor other signs of toxicity or even death in any of the rats tested during the 14-day observation period. Hence, the LD_50_ could not be determined. The compounds did not lead to any adverse reactions like increased motor activity, blinking eyes, tremors, convulsion, lacrimation, stimulation, muscle weakness, sedation, urination, salivation, lethargy, sleep, arching, rolling or coma up to a dose of 5000 mg/kg. Any test substance showing an LD_50_ of 1000 mg/kg after oral administration can be considered safe [56]. This result indicates that the compounds under study, when given orally, could be considered relatively safe. Recently, Mohan et al. [56] observed no mortality with an *A. nilotica* leaf extract on oral administration to rats at doses up to 2000 mg/kg; while Gouta et al. [57] reported in mice LD_50_ = 7393.4 mg/kg of the ethyl acetate fruit fraction. With respect to in vitro cytotoxicity, each drug tested behaved distinctly in the cell line. The distinct effects may be due to either their diversity or diverse mechanisms associated with each of the compounds. The cytotoxicity tests [58] confirm the reported result of procyanidin reducing cell viability for 15% at 127 µg/mL in Caco-2 cells. The selectivity index of almost all proanthocyanidins indicated good selective anthelminthic activity on the nematode parasite *O. ochengi* (as indicated in Table 2) given the fact that they are of cancerous origin even though they are widely use in transport studies. However, these selectivity indexes may need to be improved for further studies as much as possible according to Huggins et al. [59].

## 4. Materials and Methods

### 4.1. General Instrumentation for Phytochemical Analysis and Purification

UHPLC/+ESI-QqTOF MS analyses were carried out on a Bruker MicrOTOF-QII mass spectrometer coupled to a Dionex Ultimate 3000 RS UHPLC equipped with a Dionex Acclaim RSLC 120, C18 column (2.1 × 100 mm, 2.2 µm) with a binary gradient (A: water with 0.1% formic acid; B: acetonitrile with 0.1% formic acid) at 0.8 mL/min: 0 to 9.5 min: linear from 5% B to 100% B; 9.5 to 12.5 min: isocratic 100% B; 12.5 to 12.6 min: linear from 100% B to 5% B; 12.6 to 15 min: isocratic 5% B. The injection volume was 2 µL. UV/DAD detection was performed over a wavelength range of 200 to 400 nm. Mass spectrometric detection was performed with a Bruker Daltonics MicrOTOF-QII time-of-flight mass spectrometer equipped with an Apollo electrospray ionization source in positive mode at 5 Hz over a mass range of *m*/*z* 50 to 1000 using the following instrument settings: nebulizer gas nitrogen, 5 bar; dry gas nitrogen, 9 L/min, 220 °C; capillary voltage 4500 V; end plate offset −500 V; transfer time 70 µs; collision gas nitrogen; collision energy and collision RF settings were combined to each single spectrum of 1000 summations as follows: 250 summations with 20% base collision energy and 130 Vpp + 250 summations with 100% base collision energy and 500 Vpp + 250 summations with 20% base collision energy and 130 Vpp + 250 summations with 100% base collision energy and 500 Vpp. Base collision energy was 50 eV for precursor ions with a *m*/*z* less than 500 and then linearly interpolated against *m*/*z* up to a maximum of 70 eV for precursor ions with an *m*/*z* of up to 1000. Internal dataset calibration (HPC mode) was performed for each analysis using the mass spectrum of a 10 mM solution of sodium formate in 50% isopropanol that was infused during LC re-equilibration using a divert valve equipped with a 20-µL sample loop. Sample concentrations: the crude extract was injected at a concentration of 5 mg/mL, fractions at 0.5 mg/mL.

Analytical HPLC UV-DAD was performed with a Jasco (Groß-Umstadt, Germany) HPLC system with autosampler AS-2055 plus, ternary pump PU-2055 plus, degasser DG-2080-54, column oven Jetstream plus, set at 25 °C, UV-DAD-detector: MD-2010 plus, Software “Chrompass” for instrument control and data processing. A Macherey & Nagel (Düren, Germany) Nucleodur RP-18 column (125 × 4.6 mm, 5 µm, endcapped) with a column guard was used. Water (aqua Millipore) (A) with 0.1% TFA: acetonitrile (B) with 0.1% TFA gradient was used at a flow rate of 1 mL/min; gradient: 0 min: 95% (A), 50 min: 30% (A), 52 min: 20% (A), 55 min: 0% (A); 60 min: 0% (A); re-equilibration: 10 min; injection volume: 10 µL. Samples were dissolved in MeOH (HPLC-grade) with a concentration of 1 mg/mL.

Preparative HPLC separations were performed on a Jasco (Groß-Umstadt, Germany) prep. HPLC system (pump: PU-2087 plus; diode array detector MD 2018 plus; column thermostat CO 2060 plus; autosampler AS 2055 plus; LC Net II ADC Chromatography Data Solutions; sample injection loop: 2000 µL) on a Reprosil 100 C-18 column (5 µm, 250 × 20 mm) column with a binary gradient: water with 0.1% trifluoroacetic acid (A); acetonitrile with 0.1% trifluoroacetic acid (B) at 15 mL/min with: 0 to 5 min: linear from 5% B to 25% B; 5 to 10 min: linear 25% B to 30% B; 10 min to 35 min: linear from 30% B to 70% B; 35 min to 38 min: linear 70% B to 100% B; 30 min to 35 min: isocratic 100% B; injection volume: 283 µL. Samples were dissolved in MeOH (HPLC-grade) with a concentration of 10 mg/mL.

NMR spectra were acquired at 303 K in methanol-*d*_4_ on an Agilent DD2 600 spectrometer (Santa Clara, CA, USA), operating at 14.1 Tesla, observing ^1^H and ^13^C at 600 and 150 MHz.

### 4.2. Chemicals and Plant Material

*Acacia nilotica* fruits, used in this study, were harvested in January 2012 from the Far-North regions of Cameroon (GPS: 10°61′24.1″ N and 14°36′08.4″ E at an altitude of 392 m above sea level). Preliminary identification was made by botanists of the Department of Biological Sciences, University of Ngaoundere, Cameroon. A voucher specimen was deposited in the National Herbarium of Yaounde (Voucher Number 8582HNC).

Epicatechin-3-*O*-gallate was purchased from Sigma (Deisenhofen, Germany). Samples of (+)-gallocatechin; (−)-epigallocatechin and (−)-epigallocatechin-3-*O*-gallate, were kindly provided by F. Petereit, IPBP (Institute of Pharmaceutical Biology and Phytochemistry) University of Münster; gallocatechin was originally isolated from *Cistus albidus* [60]; epigallocatechin and epigallocatechin-3-*O*-gallate were isolated from *Cistus salvifolius* [61]. If not stated otherwise, all other chemicals were purchased from Sigma (Deisenhofen, Germany).

### 4.3. Extraction, Bioassay-Guided Fractionation and Isolation of Catechin-3-O-Gallate

#### 4.3.1. Hydro-Alcoholic Extraction from *A. nilotica* Fruits

The collected plant material was air-dried for three weeks at room temperature (25 to 28 °C), powdered, then sieved (size: 1 mm) before extraction. The air-dried and powdered fruits (500 g) were extracted with 5 L of ethanol:water (60:40 *v*/*v*) for 48 h at room temperature. The extract was filtered using Whatman No. 1 filter paper. The extraction was repeated three times, and the ethanol was removed using a rotary evaporator under reduced pressure at 40 °C. The aqueous solution was frozen and lyophilized to obtain a hydro-alcoholic extract. In this way, 142.9 g of crude hydro-alcoholic extract were obtained (yield: 35.65% *w*/*w*). Five milligrams were submitted to biological tests.

#### 4.3.2. Fractionation of Hydro-Alcoholic Extract of *A. nilotica* Fruits

Four (4) grams of the hydro-alcoholic extract of *A. nilotica* were chromatographed over silica gel 60 (300 g) using a glass column (90 cm high and 5 cm in diameter). The column was packed with a silica gel slurry prepared in hexane: ethyl acetate (EtOAc) (70:30). The sample was mixed with silica, dried and carefully introduced on top of the column. The elution was initiated with hexane: ethyl acetate (70:30) followed by mixtures of increasing EtOAc concentration 50:50, 40:60, 20:80 up to 10:90 (250 mL were used for elution in each step). The column was finally washed with 200 mL of methanol. The sub-fractions were pooled together after TLC control (20 cm × 20 cm silica gel 60 F_254_, 1 mm, elution with hexane:ethyl–acetate (1:1)) and 16 fractions were obtained: Fr. 1 (8.4 mg), Fr. 2 (3.0 mg), Fr. 3 (20.2 mg), Fr. 4 (28.7 mg), Fr. 5 (49.5 mg), Fr. 6 (35.7 mg), Fr. 7 (70.2 mg), Fr. 8 (70.9 mg), Fr. 9 (62.9 mg), Fr. 10 (48 mg), Fr. 11 (51.2 mg), Fr. 12 (244.5 mg), Fr. 13 (182.8 mg), Fr. 14 (58.8 mg), Fr. 15 (2.7 mg) and Fr. 16 (51.8 mg). 5 mg of each fraction were tested for anthelmintic activity against *C. elegans*. The most active fractions (8, 9, 10) were subjected to ultra-high performance liquid chromatography/mass spectrometry (UHPLC/+ESI-QqTOF MSMS) and high performance liquid chromatography (HPLC) to obtain chromatographic profiles.

The high resolution +ESI MS and MSMS data of the active fractions indicated the presence of catechin and gallocatechin derivatives by the characteristic molecular ions and fragmentation (see Figure 1 and Figure 2). From Fraction 8, the main constituent catechin-3-*O*-gallate was isolated by prep HPLC. The isolated compound was identified unambiguously by 1D and 2D-NMR spectroscopic measurements. All data were in agreement with the literature [31,32].

### 4.4. Nematocidal Activity of the Pure Compounds

#### 4.4.1. *Caenorhabditis elegans* Strains and Monoxenic and Axenic Culture Conditions

The following *C. elegans* strains were used: N2 Bristol, referred to as wild type (WT); levamisole-resistant strains ZZ16 (*lev-9*(x16) X), CB211 (*lev-1*(e211) IV), the albendazole-resistant strain CB3474 (*ben-1*(e1880) III) and ivermectin-resistant strains VC722 (*glc-2*(ok1047) I) and DA1316 (*avr-14*(ad1302) I *avr-15*(ad1051) *glc-1*(pk54)). All strains were obtained from the Caenorhabditis Genetic Centre (CGC, Minneapolis, MN, USA). Strains were maintained at 20 °C on nematode growth medium (NGM) [62]. All worms were age-synchronized and obtained from eggs that were isolated by bleaching [63]. Aged-synchronous worms were then successfully grown in axenic medium ((3% (*w*/*v*) yeast extract, 3% (*w*/*v*) soy peptone, 1% (*w*/*v*) glucose, 0.5 mg/mL cholesterol and 0.5 mg/mL bovine hemoglobin) supplemented with 100 U/mL penicillin and 100 µg/mL streptomycin modified from the semi-define aqueous medium used by Chitwood and Feldlaufer [64] and incubated at 20 °C for worms to developed to L4 (or young adult stage). Due to the lack of an external food source, worms grew rather slowly at 20 °C [65,66].

#### 4.4.2. In Vitro Screening: Lethality Testing Using *Caenorhabditis elegans*

Young adult (L4 stage) worms were used to assess drug lethality. Monitored endpoint was 48 h mortality. Tests were performed in 24-well plates. Five hundred (500) microliters of test solution, which is M9-buffer (3 g KH_2_PO_4_, 6 g Na_2_HPO_4_, 5 g NaCl, 0.25 g MgSO_4_.7H_2_O, in 1 L of water after been autoclaved supplemented with 2% glucose, 0.5% cholesterol), were distributed in 24-well plates. The volume of M9-buffer equivalent to the drug to be added for each drug final concentration (in each test, three to six concentrations were used) was removed. Untreated worms in M9-buffer were used as negative control. Positive controls were treated with levamisole, albendazole and ivermectin. Tested worms were treated with CE, fractions and pure compounds. Crude extract fraction (stock solution at 100 mg/mL) and pure compound (stock solution at 100 mg/mL) previously dissolved in M9-buffer were then added in well plates at increasing concentration (varying from 0 to 1000 or 1500 µg/mL for extract and fractions; 0 to 100 or 150 µg/mL). The young adult (10 to 15 ± 2) from the axenic culture (without food source) were then added at the test solution. The assay was incubated at 20 °C, and they were observed at each 24 h under binocular microscope for lethality determination. Dead and live worms were recorded 48 h post-exposure, then LC_50_ (lethal concentration of the extract or pure compound required to kill 50% of worms) was determined. Treatments were replicated three times. Worm mortality was investigated under microscope and worms was scored as dead if they were immotile, lacking movement following shaking or if they failed to move upon prodding with a worm pick and were fully elongated [66]. *C. elegans* with LC_50_ < 30 µg/mL of drug is highly sensitive; 30 < LC_50_ < 50 µg/mL is moderately sensitive; and 50 µg/mL < LC_50_ is less sensitive.

#### 4.4.3. *Onchocerca ochengi* Isolation and Culture Conditions

• Male worms:

The adult male worms were isolated as previously describe by Ndjonka et al. [16] from fresh pieces of cattle skin (cow-udder) with palpable nodules obtained from the communal slaughter house of Ngaoundere in the Adamawa region of Cameroon. Nodules removed from the skin were brought to the laboratory, washed, drained and sterilized with 70% ethanol for dissection. After dissection of nodules, *O. ochengi* were extracted, isolated and washed three times in sterile phosphate-buffered saline (PBS). To analyze the anthelmintic activity of extract, fractions and pure compounds, the worms were incubated at 37 °C.

• Microfilaria (Mf):

Microfilariae were isolated as describe by Cho-Ngwa et al. and Ndjonka et al. [14,17]. About 10 skin snips were obtained from different locations of the skin. The skin snips were then incubated at 37 °C in PBS to let Mf emerge. The highly motile Mf were concentrated by centrifugation at 400× *g* for 10 min and quantified [22]. The Mf were incubated at 37 °C with the crude extract, fractions and pure compounds.

• Female worms:

The adult female worms were isolated by digestion of the nodules with collagenase at 37 °C. The female worms were cultured in a CO_2_-incubator [67]. For anthelmintic activity, they were incubated with the crude extracts, fractions and pure compounds.

#### 4.4.4. In Vitro Assays: Lethality of *Onchocerca ochengi*

Adult worms (six individuals per well for males and one per well for the female) and Mf were washed twice and were subsequently transferred to RPMI-1640 medium supplemented with l-glutamine, 100 U/mL penicillin and 100 µg/mL streptomycin and incubated at 37 °C in 24-well plates (96-well plate for Mf) with different concentrations of the drugs tested. Worm mortality was checked by observation under the binocular microscope after 48 h [14,16,17]. After shaking, immotile and fully-elongated worms were considered to be dead. All tests were done in three independent triplicate determinations.

### 4.5. In Vitro Cytotoxicity and CC_50_ Determination

Cell culture: Cacao-2 cells at Passage 70 were kindly provided by Prof. Hensel, Institute of Pharmaceutical Biology and Phytochemistry, University of Münster, and were cultured as previously describe by Zumdick et al. [58]. Briefly, the cells from Passages 70 to 82 were grown in 75 cm^2^ culture flasks in Dulbecco’s Modified Eagle Medium (DMEM) supplemented with 4.5 g/L d-glucose, 2.2 g/L NaHCO_3_, 10% FBS, 1% non-essential amino acids, 100 IU/mL penicillin and 100 µg/mL streptomycin in an atmosphere of 5% CO_2_ and 95% relative humidity at 37 °C.

Cytotoxicity test: The cytotoxicity assays were performed according to the method of Rahman et al. [38]. Briefly, 1.5 × 10^4^ viable cells/well were seeded in a 96-well plate and incubated for 24 to 48 h. Then, to initiate the experiments, the culture medium was replaced by fresh supplemented with drugs (increasing concentrations up to 100 or 150 µg/mL) dissolved in the medium for the tests. DMEM samples were used as the negative control. After 72 h of incubation, test solution was replaced by 30 µL of a 5 mg/mL 3-(4,5 dimethylthiazol-2-yl)-2,5-diphenyl tetrazolium bromide (MTT) solution in each well and incubated at 37 °C in a 5% CO_2_ atmosphere with 95% humidity incubator for 4 h. The medium was removed, and formazan, generated by the activity of dehydrogenases, was solubilized with 70 µL of acidified isopropanol (0.4 M HCl). The amount of MTT-formazan that is directly proportional to the number of living cells was determined by measuring the optical density (OD) at 540 nm using a spectrophotometer. All controls used were treated equally. As control and to determine 100% growth, Caco-2 cells were grown in the absence of drugs so as to calculate cell viability. All determinations were performed in triplicate. The concentration of the drugs that killed 50% of the cells (CC_50_ 50% cytotoxic concentration) was calculated [68]. The selectivity index (SI) values were calculated as the ratio: SI = CC_50_ of drug on Caco2/LC_50_ of drug on worm.

### 4.6. Acute Oral Toxicity on Rats

Adult Wistar rats (*Rattus norvegicus*), males aged 7 to 8 weeks and weighing 150 to 200 g, used for the experiments were obtained from LANAVET (Laboratoire National Vétérinaire, Garoua, Cameroon). The rats were acclimatized for one week in the Veterinary Research Laboratory of the Institute of Agricultural Research for Development, Wakwa, Cameroon, before initiating experiments. Animals were housed in groups of three (one group for the negative control, six groups for each drug at the first phase, six others at the second phase) under ambient temperatures (23 °C ± 1 °C) with relative humidity 55 ± 5% and 12 h light/dark cycle in polycarbonate cages. They were fed with standard rodent pellets and had unrestricted access to clean drinking water. They were monitored by a veterinarian for health status. There is no law yet regulating animal research in Cameroon [22]. All animal-related experimental procedures were approved by the regional delegation of Livestock, Fisheries and Animal industries (No.075/16/L/RA/DREPIA).

Acute oral toxicity (LD_50_, that is the dose that kills 50% of animals of the tested group) was measured using the method of the OECD guidelines for the testing of chemicals [56] following the European Community guidelines for the protection of animals used in experiments [69] and respecting the 3Rs (Replacement, Refinement, Reduction) [70]. Drugs in distilled water were orally administered separately at doses of 10 mg/kg, 100 mg/kg and 1000 mg/kg body weight. The negative controls were distilled water-administered rats. They were given to three groups of three rats each and observed for 24 h. Since no mortality was observed, the second phase was done with three groups of three rats each at drugs doses of 1500 mg/kg, 3000 mg/kg and 5000 mg/kg orally. The test was done at a single oral dose for each rat in a 5 mL/kg volume. The experimental animals were starved for 24 h before and 4 h after treatment. They were observed for 24 h; the number of deaths was recorded and the LD_50_ determined. The appearance of toxic symptoms, such as behavioral changes, locomotion, convulsions and mortality, was observed and recorded. Animals were observed constantly for the first 30 min after administration and, thereafter, every 4 h up to 24 h and, subsequently, once a day for up to 14 days.

### 4.7. Statistical Analysis

The LC_50_ values (lethal concentration of the extract or pure compound required to kill 50% of helminths) of all drugs were calculated using MS Excel. Positive controls were ivermectin, levamisole and albendazole. The CC_50_ values (50% cytotoxic concentration) of all drugs were calculated using GraphPad Prism 5.03. Data comparison was done using analysis of variances. Values were significant at *p* < 0.001.

## 5. Conclusions

Our results show that the polyphenol-rich fractions of *A. nilotica* containing mainly galloylated catechin and gallocatechin derivatives are potential anthelmintics. The reported ethnomedicinal use of this plant by traditional healers thus appears justified and receives a scientific basis. It is furthermore confirmed that the tested proanthocyanidins, most prominently ECG and EGCG, possess significant anthelmintic potency without noticeable adverse effects in animal experiments. This study provides the first evidence of a very high anti-*Onchocerca* activity of ECG and EGCG with LC_50_ values less than 2.5 µg/mL, i.e., in the same activity range as the positive controls levamisole, ivermectin and albendazole. These compounds and, possibly, further galloylated proanthocyanidins yet to be isolated from *A. nilotica* or other plant sources thus deserve further in-depth investigation as potential anthelmintics. In vivo study using the newly-validated *Onchocerca ochengi* microfilaricidal hamster model will be important to compare the similitude of the efficacy of in vitro and in vivo nematotoxicity.

## Figures and Tables

**Table 1:** LC_50_ of *Acacia nilotica* crude extract and pure proanthocyanidin derivatives at 48 h post-treatment against *O. ochengi* and *C. elegans* (wild type and drug-resistant strains).

LC_50_/48 h in μg/mL (µM)									
Worms	CE	CG	ECG	GC	EGC	EGCG	Levamisole	Ivermectin	Albendazole
Means LC_50_ ± SD									
***O. ochengi* males**	11.5 ± 0.1	7.6 ± 0.2 (17.2)	2.1 ± 0.3 (4.7)	4.2 ± 0.1 (13.6)	2.1 ± 0.4 (6.8)	1.2 ± 0.5 (2.6)	1.0 ± 1.0 (5.1)	1.2 ± 0.5 (1.3)	4.2 ± 0.3 (15.7)
***O. ochengi* females**	11.0 ± 0.2	4.5 ± 0.3 (10.2)	2.1 ± 0.6 (4.7)	5.5 ± 0.3 (18.0)	3.3 ± 0.6 (10.6)	1.0 ± 0.5 (2.3)	2.1 ± 0.1 (10.2)	1.5 ± 0.3 (1.7)	1.0 ± 0.2 (3.9)
***O. ochengi* microfilariae**	10.8 ± 0.3	4.2 ± 0.1 (9.4)	1.0 ± 0.4 (2.2)	3.2 ± 0.3 (10.4)	3.3 ± 0.5 (10.6)	1.3 ± 0.2 (2.8)	1.0 ± 0.3 (5.1)	1.5 ± 0.0 (1.7)	2.1 ± 0.5 (7.8)
***C. elegans* WT**	350 ± 1.1	90.6 ± 0.07 (204.8)	33.8 ± 2.7 (2.4)	74.3 ± 0.3 (242.4)	62.7 ± 0.2 (204.7)	49.8 ± 0.8 (108.6)	1.4 ± 0.2 (7.2)	1.2 ± 0.1 (1.3)	7.1 ± 0,2 (26.4)
***C. elegans* ZZ16**	987.9 ± 0.3	95.3 ± 0.1 (215.4)	39.5 ± 0.9 (76.4)	65.6 ± 0.2 (214.2)	92.0 ± 0.3 (301.4)	>100	>100	nd	nd
***C. elegans* CB211**	934.3 ± 1.0	96.4 ± 0.1 (217.9)	41.7 ± 1.2 (89.3)	21.5 ± 0.3 (70.2)	83.3 ± 0.6 (271.9)	26.3 ± 1.3 (57.4)	>100	nd	nd
***C. elegans* CB3474**	982.9 ± 0.6	95.2 ± 0.3 (215.2)	19.6 ± 0.8 (44.2)	83.1 ± 0.1 (271.4)	53.8 ± 0.9 (175.7)	35.7 ± 1.0 (77.9)	nd	nd	>100
***C. elegans* VC722**	985.5 ± 0.7	91.8 ± 0.3 (207.5)	27.2 ± 1.4 (61.4)	96.3 ± 0.07 (314.3)	93.8 ± 0.3 (306.3)	43.7 ± 2.1 (95.4)	nd	>100	nd
***C. elegans* DA1316**	999.3 ± 1.2	96.3 ± 0.3 (217.7)	27.4 ± 1.1 (61.9)	82.5 ± 0.1 (269.4)	87.5 ± 0.5 (285.7)	40.8 ± 0.3 (89.1)	nd	>100	nd

Each value represents mean ± SD (standard deviation); values between brackets are LC_50_ after 48 h calculated in µM; nd = not determined; CE: crude extract; CG: catechin-3-*O*-gallate; ECG: epicatechin-3-*O*-gallate; GC: gallocatechin; EGC: epigallocatechin; EGCG: epigallocatechin-3-*O*-gallate.

**Table 2:** Cytotoxicity activity of pure compounds and crude extract from *A. nilotica* fruits and positive controls on Caco-2 cell lines. Selectivity index (SI) on *C. elegans* wild-type, *O. ochengi* males, females and microfilariae.

Compounds	Cytotoxicity μg/mL (μM)	Selectivity Index (SI)			
	Means CC_50_ ± SD				
	Caco-2	*C. elegans*	*O. ochengi*		
		Wild Type	Males	Females	Microfilariae
**CE**	93.2 ± 1.1	0.3	8.1	8.5	8.6
**CG**	66.3 ± 0.6 (149.9)	0.7	8.7	14.7	15.8
**ECG**	67.6 ± 0.5 (152.8)	2.0	32.2	32.2	67.6
**GC**	66.7 ± 0.7 (217.8)	0.9	15.9	12.1	20.8
**EGC**	47.1 ± 0.5 (153.8)	0.8	22.4	14.3	14.3
**EGCG**	60.9 ± 0.8 (132.9)	1.2	50.8	60.9	46.9
**Levamisole**	27.3 ± 0.8 (31.2)	18.8	27.3	13.0	27.3
**Ivermectin**	28.7 ± 0.7 (140.5)	23.9	23.9	19.1	19.1
**Albendazole**	29.4 ± 0.5 (110.8)	4.1	7	29.4	14

Each value of cytotoxicity represents mean ± SD (standard deviation); values between brackets are CC_50_ after 48 h calculated in µM. Selectivity index (SI) = CC_50_ on mammalian cells/LC_50_ on worm.

## Supplementary Materials

Supplementary materials are available online.
