# Supplementary material for: Anti-Onchocerca and Anti-Caenorhabditis Activity of a Hydro-Alcoholic Extract from the Fruits of Acacia nilotica and Some Proanthocyanidin Derivatives
Source: Molecules. 2017 May 6;22(5):748. doi: 10.3390/molecules22050748 (PMC6154738; doi:10.3390/molecules22050748)
Supplement: Supplementary file 1 [file molecules-22-00748-s001.pdf]

Supplementary Materials: Anti-*Onchocerca* and Anti-*Caenorhabditis* Activity of a Hydro-Alcoholic Extract from the Fruits of *Acacia nilotica* and Some Proanthocyanidin Derivatives

Jacqueline Dikti Vildina [1], Justin Kalmobe [1], Boursou Djafsia [1], Thomas J. Schmidt [2], Eva Liebau [3] and Dieudonne Ndjonka [1,*]

Table S1. LC_{50} of *Acacia nilotica* fractions at 48 h post-treatment against *C. elegans* wild type.

Fractions	LC_{50} (µg/mL)
Acacia nilotica Fraction 1	>1000
Acacia nilotica Fraction 2	>1000
Acacia nilotica Fraction 3	>1000
Acacia nilotica Fraction 4	>1000
Acacia nilotica Fraction 5	>1000
Acacia nilotica Fraction 6	>1000
Acacia nilotica Fraction 7	962.1 ± 0.5
Acacia nilotica Fraction 8	73.8 ± 0.2
Acacia nilotica Fraction 9	70.1 ± 0.3
Acacia nilotica Fraction 10	50.6 ± 0.9
Acacia nilotica Fraction 11	595.6 ± 0.8
Acacia nilotica Fraction 12	580.2 ± 0.9
Acacia nilotica Fraction 13	432.2 ± 0.6
Acacia nilotica Fraction 14	916.1 ± 0.9
Acacia nilotica Fraction 15	922.2 ± 0.9
Acacia nilotica Fraction 16	910.6 ± 0.4

Each value represents mean ± SD.

Table S2. Results of acute toxicity on rats of pure compounds and crude extract from *A. nilotica* fruits.

Mortality of Rate Rats: Rat Number End/Start							
		Phase 1 (mg/kg)			Phase 2 (mg/kg)		
**Compounds**	**Control**	**10**	**100**	**1000**	**1500**	**3000**	**5000**
**CE**	0/3	0/3	0/3	0/3	0/3	0/3	0/3
**CG**	0/3	0/3	0/3	0/3	0/3	0/3	0/3
**ECG**	0/3	0/3	0/3	0/3	0/3	0/3	0/3
**GC**	0/3	0/3	0/3	0/3	0/3	0/3	0/3
**EGC**	0/3	0/3	0/3	0/3	0/3	0/3	0/3
**EGCG**	0/3	0/3	0/3	0/3	0/3	0/3	0/3
**Albendazole**	0/3	0/3	0/3	0/3	0/3	0/3	0/3
